# New Insights on *Leptospira* Infections in a Canine Population from North Sardinia, Italy: A Sero-Epidemiological Study

**DOI:** 10.3390/biology10060507

**Published:** 2021-06-07

**Authors:** Ivana Piredda, Maria Nicoletta Ponti, Angela Piras, Bruna Palmas, Pierangela Pintore, Aureliana Pedditzi, Valentina Chisu

**Affiliations:** 1 Istituto Zooprofilattico Sperimentale della Sardegna, Department of Animal Health, Laboratory of Seroimmunology, 07100 Sassari, Italy; nicoletta.ponti@izs-sardegna.it (M.N.P.); bruna.palmas@izs-sardegna.it (B.P.); pierangela.pintore@izs-sardegna.it (P.P.); aureliana.pedditzi@izs-sardegna.it (A.P.); valentina.chisu@izs-sardegna.it (V.C.); 2 Azienda Socio Sanitaria Locale, Azienda Tutela Salute, 09047 Cagliari, Italy; ang.piras@atssardegna.it

**Keywords:** *Leptospira*, dogs, rats, seroprevalence, canine, MAT, MLST, vaccines, zoonosis

## Abstract

**Simple Summary:**

Leptospirosis is a widespread zoonosis caused by pathogenic spirochaetes of the genus *Leptospira*. Pathogenic leptospires live in the kidneys of different mammalian species and are excreted in the environment with the urine. The infection of humans is mainly caused by direct contact with infected animals or indirectly by contact with a contaminated environment. The aim of this study was to perform a sero-epidemiological survey to assess the presence of antibodies against *Leptospira* serovars in a representative sample of kennel and owned dogs from a selected area of Sardinia Island. In addition, kidney homogenates from rodents collected from the same study area were also analyzed by culture-based and real-time PCR-based testing methods. Higher antibody titers were observed against *Leptospira* Icterohaemmorrhagiae, which is believed to be responsible for the majority of severe cases of leptospirosis in humans. The detection of pathogenic *Leptospira* serotypes in dogs from northern Sardinia may represent a potential risk of infection for humans and contribute to the spread of the bacteria in the environment. Public health strategies to control possible *Leptospira* outbreaks should be implemented to prevent the disease from becoming a major medical and veterinary problem in this region.

**Abstract:**

Leptospirosis is a widespread zoonosis recognized as a re-emerging infectious disease in a wide variety of animal species, including humans and dogs. No data exist regarding the presence of *Leptospira* species in the canine population of Sardinia Island. This study reports the first sero-survey for leptospirosis in kennel and owned dogs from six areas of the north of Sardinia. Sera from 1296 dogs were tested by microscopic agglutination test (MAT) specific for nine different serovars that are known to be well widespread in the Mediterranean environment. Moreover, kidney homogenates from rodents collected from the study area were also analyzed by LipL32 real-time PCR and multi-locus sequence type (MLST) on the basis of the analysis of seven concatenated loci. A total of 13% of the examined sera (95%CI: 11–15) tested positive for one or more serovars of *Leptospira* MAT detected; antibodies for serogroup Icterohaemorrhagiae (57%; 95%CI: 49–65) were the most common, followed by serovars Bratislava (22%; 95%CI: 16–28), Canicola (14%; 95%CI: 9–19), and Grippotyphosa (7%; 95%CI: 3–11). MLST analyses on isolates from rodents identified *L. interrogans* and *L. borgpetersenii* genomospecies. Different serovars belonging to pathogenic *Leptospira* serogroups are circulating in dogs from the island. Moreover, data obtained from rodents, indicated that rodents likely act as reservoir of spirochetes. Further sero-epidemiological studies are needed in order to obtain data from other collection sites in Sardinia and to increase the information on *Leptospira* species circulating in this area.

## 1. Introduction

Leptospirosis is a cosmopolitan zoonosis caused by bacteria belonging to the genus *Leptospira*, which is well known for infecting humans and a wide variety of domesticated and wild vertebrates [1]. *Leptospira* species have been described also in several ‘unconventional’ hosts [2] such as reptiles [3], amphibians [4], and cetaceans [5,6], as well as from many other different orders, suggesting that most microbial diversity in hosts is largely unknown. Humans can become infected by direct exposure to infected animals and their products (urines or body fluids) [7]. Canine leptospirosis due to pathogenic *Leptospira* species has been described on almost every continent [8,9,10]. Dogs are known to be reservoir hosts for *L. interrogans* serovar Canicola; therefore, shedding a huge amount of leptospires in urine can be expected without initiation of an appropriate antibiotic therapy [11,12,13]. It is increasingly recognized that dogs can also shed other *Leptospira* serovars or species in the absence of clinical signs, raising concerns for zoonotic transmission [14,15]. In dogs, *Leptospira* infection is usually associated with activities that include drinking from an infected water sources, swimming in contaminated water, or eating food that has been exposed to contaminated water or potentially infected by rodents or others carrier wildlife [16]. Since dogs act as bridge between wildlife and humans, they could be used as a useful indicator of the presence and distribution of the bacteria in specific areas [17,18]. However, since *Leptospira* does not grow easily with the use of standard culture techniques, specific indirect tools have been mainly used for diagnosis [19]. Microscopic agglutination test (MAT) is the gold standard method for testing *Leptospira* infection, even if cross-reaction between different *Leptospira* serovars could complicate the diagnosis [7,20]. Currently, direct detection of *Leptospira* DNA by real-time PCR and genotyping by multi-locus sequence typing (MLST) from various clinical samples allows for the establishment of the presence of the pathogen [19,21]. The aim of this study was to carry out a sero-epidemiological investigation in order to evaluate the presence of antibodies against *Leptospira* serovars in a representative sample of stray and non-stray dogs from a specific area of the island of Sardinia. The molecular detection and characterization of *Leptospira* strains from wild rodents collected from areas near those of study was also performed.

## 2. Materials and Methods

### 2.1. Ethical Statement

Animal experiments carried out in this study were approved by the ethical committee of the Istituto Zooprofilattico Sperimentale della Sardegna (IZS) and further authorized by the Italian Ministry of Health (Ministero della Salute) in accordance with Council Directive 2010/63/EEC of the European Union and the Italian D.Igs 26/2014 (protocol 1248/2015-PR).

### 2.2. Study Design and Sample Collection

**Figure 1:**
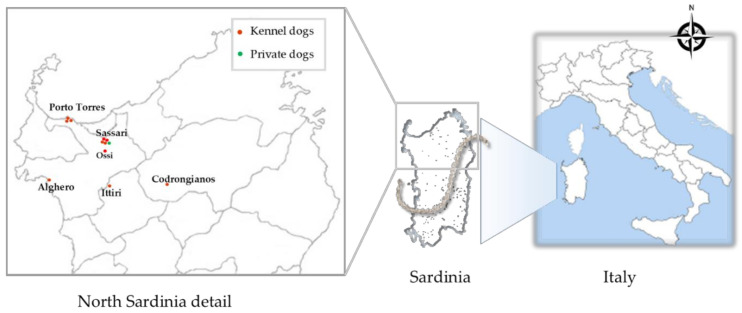
Geographical distribution of the 11 kennels analyzed in this study (red dot) and of the owned dogs (green dot).

Between November 2016 and March 2018, a cross sectional study was conducted in order to investigate the presence of *Leptospira* in dogs from 11 kennels located in different sites of North Sardinia (Figure 1). A database including information about dog species, gender, age, size, and collection sites of kennels was created for each animal. The microchip number, the last vaccination date and presence or absence of any physical signs were also evaluated.

Moreover, blood samples from 88 client-owned dogs presented for routine vaccination or health assessment at veterinary clinics located mainly in the area of Sassari city were included in this study, as shown in Table 1. Dog owners signed a consent of availability and acceptance to include their dogs in this study. For each participating dog, place of origin, gender, age, household variables (indoor/outdoor or strictly indoor), breed, and history of vaccination were recorded. All dogs underwent to a clinical examination in which body condition, color of mucous membranes, temperature, and clinical signs of leptospirosis were evaluated. Blood samples were collected from each dog and stored at 4 °C until serum separation (obtained within 24 hours after centrifugation). Serum was also frozen at 20 °C until further serological and molecular analyses. From a total of 18 dogs casually selected, urine samples were obtained by cystocentesis and sent to IZS laboratories for *Leptospira* detection. In order to obtain a representative sample of the rodent population, we placed snap and/or mechanical traps in each kennel identified for this study and investigated them daily (Table 1). When the rodents were captured, they were transported to the IZS and euthanized using the pentobarbital IP euthanasia technique [22] and morphologically classified by an experienced veterinarian [23]. Blood samples were obtained by intracardiac puncture (*n* = 17). Kidney and liver tissues were also collected from 237 rats and included in this study. Where possible, urine samples were also obtained (*n* = 4).

### 2.3. Microscopic Agglutination Test

An aliquot of serum was examined by MAT following the recommendation performed by National Reference Centre of Leptospirosis (IZSLER, Brescia, Italy). Live strains of eight 7–10 days of culture of *Leptospira* species belonging to nine different serogroups (most frequently found from Mediterranean area) were used (Table 2). The density of leptospires was assessed using a counting chamber (Petroff-Hausser, USA) and adjusted to 2 × 10^8^ leptospires/mL.

All sera were diluted using phosphate buffered saline (PBS) (pH 7.6) at 10^−2^ dilution, mixed individually with each serovar suspension in the proportion 1:1, and incubated at 29 °C for 2 h. Pure PBS solution was used as negative control. The analysis was performed in dark field microscopy (Olympus BX50; Olympus Corp., Tokyo, Japan) with magnification of 100×. Samples were considered positive when 50% or more leptospires were agglutinated at 10^−2^ dilution, considering a cut-off titer of 100. The reactive serovar was considered the one that presented the highest titer. Positive samples were further diluted in the proportions 1:200, 1:400, 1:800, and 1:1600, and tested only to the reacted serovar to determine their titer.

### 2.4. Culturing of Leptospires

Ellinghausen–McCullough–Johnson–Harris (EMJH) semi-solid and liquid medium was used for the isolation of *Leptospira* from urine and organs. 5-Fluorouracil (5-FU) was added due to minimize bacterial contamination. Samples of kidney and liver were homogenized as previously described [24]. A total of 500 µL of urine (500 µL diluted 1:100 in PBS) and 25 mg of tissue were suspended in EMJH–fluorouracil medium at 28 °C and cultured for a period of three months. The media were examined under dark-field microscopy for the presence of leptospires approximately every seven days. Pure isolates, free of contaminants, were used for further molecular identification.

### 2.5. DNA Extraction and PCR Assays

Blood, homogenized organs, urine samples, and isolates were extracted using the DNeasy Blood and Tissue Kit^®^ (Qiagen, Hilden, Germany), according to the manufacturer’s instructions. The final elution volume reduced to 50 µL was the unique modification performed on the original protocol. All DNA samples were then screened for detection of pathogenic *Leptospira* by real-time PCR (qPCR) assay with primers LipL32-45F (5′-AAGCATTACCGCTTGTGGTG-3′) and LipL32-286R (5′-GAA CTCCCA TTT CAG CGA TT-3′), and the probeLipL32-189P (FAM-5′-AA AGCCAG GAC AAG CGCCG-3′-BHQ1), targeting a fragment of the *lipL32* gene [25]. PCR reaction mix composition and PCR temperature cycling conditions were the same as those used in a previous study [24]. A negative control (DNA extracted from water) and a positive control (DNA extracted from the reference strain of *L. interrogans* ATCC^®^ BAA1198D5TM) were included in each run.

### 2.6. Multi Locus Sequence Types (MLST)

In order to reveal sequence types (STs) of *Leptospira* isolates, we performed MLST assay using the 7 housekeeping genes *pntA*, *sucA*, *tpiA*, *pfkB*, *mreA*, *glmU*, and *caiB* proposed by Boonsilp in 2013 [26]. Each allele and the allelic profiles (glmU-pntA-sucA-tpiA-pfkB-mreA-caiB) were submitted to the *Leptospira* database (http://pubmlst.org/leptospira accessed on 10 May 2021) to define the STs.

## 3. Results

A total of 1296 dogs were tested for leptospirosis in this study. Among them, kennel dogs were 1208 (93%; 95%CI: 92–94) and owned dogs were 88 (7%; 95%CI: 6–8) in number (Table 3). The examined dogs did not show any apparent clinical signs related to Leptospira infection. The dogs were classified on the basis of sex as female (51%; 95%CI: 48–54) and male dogs (49%; 95%CI: 46–52). With regards to age, 15% of dogs were under 2 years old (95%CI: 13–17), 52% were between 2 and 8 years (52%; 95%CI: 49–55), and 33% were over 8 years (95%CI: 30–36). Regarding the size of the dogs, the percentages of 10% (95%CI: 8–12), 86% (95%CI: 84–88), and 4% (95%CI: 3–5) were attributed to dogs of small, medium, and large size, respectively. Of the 1296 tested dogs, 88% (95%CI: 86–90) were vaccinated using a bivalent vaccine including serovars Icterohaemorrhagiae and Canicola. A total of 12% (95%CI: 10–14) were not immunized.

**Figure 2:**
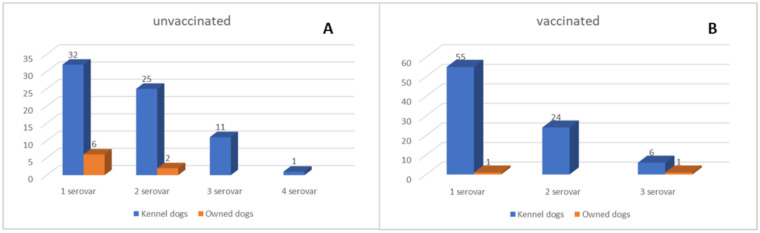
Graphic representations of the single and multiple seropositivity for one or more Leptospira serovars detected in vaccinated (**A**) and unvaccinated dogs (**B**).

From a total of 1296 dog sera examined by MAT, 164 (13%; 95%CI: 11–15) tested positive for one or more serovars of Leptospira (Figure 2A). Specifically, the prevalence rate was of 89% (*n* = 154; 94%CI: 90–98) in kennel dogs and 11% (*n* = 10; 6%; 95%CI: 2–10) in owned dogs (Table 4). In Table 4, dogs with confirmed Leptospira diagnosis are classified on the basis of the different variables (sex, age, size, and vaccination status). Female and male positive dogs were 80 (49%) and 84 (51%) in number, respectively. MAT-positive results were more frequently diagnosed in adult dogs (93%; 95%CI: 89–97) as compared with young animals (7%; 95%CI: 3–11), and 67% of medium-sized dogs confirmed the presence of Leptospira infection.

Positive samples included 77 (47%; 95%CI: 39–55) non-vaccinated dogs (including those with no history of recent vaccination) and 87 (53%; 95%CI: 45–61) dogs that received the vaccine from less than six months since the blood collection. Among the non-vaccinated dogs, 38 (49%; 95%CI: 38–60), 27 (35%; 95%CI: 24–46), 11 (4%; 95%CI: 6–22), and 1 (2%; 95%CI: 0–5) showed MAT positivity for one, two, three, and four serovars, respectively (Figure 2A). Copenhageni and Icterohaemorrhagiae (61%; 95%CI: 50–72) were the most common serovars, followed by Bratislava (17%; 95%CI: 9–25), Grippothyphosa, and Canicola (both 8%; 95%CI: 2–14). Titers ranging from 1:100 to 1:400 were the most commonly detected (Table 5).

Positive results associated with dogs recently vaccinated are listed in Table 5. A total of 56 (64%; 95%CI: 54–74), 24 (28%; 95%CI: 19–37), and 7 (8%; 95%CI: 2–14) dogs showed positivity for one, two, and three serovars, respectively (Figure 2B). Antibody titers for Copenhageni were 1:100 and 1:1400 in 52% (95%CI: 42–62) and 2% (95%CI: 0–5) of the vaccinated dogs tested. One dog had titers of 1:3200 for Icterohaemorrhagiae serovar, which resulted in the second most common serovar detected.

All of the 21 urine samples collected from dogs (*n =* 18) and rats (*n =* 4) were negative when the lipL32 gene qPCR specific for pathogenic leptospires was used. Of 237 rodent organs tested, Leptospira DNA was obtained from 28 (12%; 95%CI: 8–16) kidneys and from 7 (3%; 95%CI: 1–5) cultured isolates. MLST analysis (Table 6) by using the database generated for the 7-locus scheme (http://pubmlst.org/leptospira 10 May 2021) highlighted that three different STs were obtained, namely, ST17 from one *Rattus rattus*, ST149 from two *Rattus rattus* and three *Apodemus sylvaticus*, and ST198 from one *Rattus norvegicus* (Table 6).

## 4. Discussion

This work represents the first cross-sectional study in which the presence of *Leptospira* serovars was evaluated in Sardinian dogs by microscopic agglutination test (MAT). The 13% of examined dogs (154/1208) showed sero-reactivity to five of the nine *Leptospira* serovars of the MAT panel used herein. Despite the fact that MAT is considered to be the reference method for the serodiagnosis of leptospirosis both in humans and animals, including dogs [20], this test suffers from bias related to non-specific cross reactions, such as those linked to a recent dog vaccination [27].

Among the 26 dogs that did not receive any vaccine at all and the 51 with no history of recent vaccination, the serovar Bratislava, Icterohaemorrhagiae, and Copenhageni were prevalent and MAT titers ranged from 1:100 to 1:400. Canicola and Grippotyphosa were also obtained at low titer, indicating a possible transmission through contaminated shared environment. One dog showed higher antibody titer of 1:1600 for Bratislava serovar, which is commonly associated with disease in domestic animals, in particular cats [28], pigs [29,30], horses [31,32], and dogs [33,34]. One 8-year-old dog with no history of recent vaccination had a double *Leptospira* co-infection showing high titers both for Copenhageni (1:1600) and Icterohaemorrhagiae (1:3200) serovars, indicating that vaccines do not ensure an immunity protection one year after vaccination [35,36]. Although dogs of all ages can be infected with *Leptospira*, in this study, only 12 dogs younger than 2 years (7%) were positive after MAT analyses. These results were in accordance with other studies [37,38,39], where the rate of positives in younger exemplars was related to better immunologic protection as a result of puppy vaccinations and maternally acquired antibodies. Icterohaemorrhagiae and Copenhageni serovars are considered to be the most representative and virulent strains of the Icterohaemorrhagiae serogroup and are typically responsible for the majority of severe cases of leptospirosis in humans [40,41]. In Italy, Icterohaemorrhagiae and Copenhageni serovars (serogroup Icterohaemorrhagiae) have been reported from several domestic mammals, including dogs [27,42,43]. *Rattus* species are considered the main reservoir for both serovars [41], suggesting a participation of these hosts in the environmental persistence of the bacterium. The large number of infected animals resulted positives for the different serovars could be also correlated with the location of kennels in rural setting populated by wild rodents (as also demonstrated by the high number of rodents collected in these areas). Thus, according to other studies, the widespread circulation of the bacterium is related to the close proximity to rural areas, making transmission easier than it is elsewhere [44,45,46]. The 12% of non-vaccinated dogs also tested seropositive for Bratislava and Canicola serovars, probably indicating the widespread circulation of these strains in the territory, associated with the low level of immune protection in this population. In humans, severe leptospirosis is most frequently associated with serogroup Icterohaemorrhagiae, in particular serovar Copenhageni [7,47]. Antibodies to *Leptospira interrogans* serovar Bratislava have been detected in dogs [33,48]; humans [49]; horses from Italy [32]; and in hedgehogs, which are indicated as potential reservoirs leading to canine infection in France and Ireland [30,50]. Similar data have been reported in dogs from Spain, where seropositive sera (MAT ≥1:100) to serovars Icterohaemorrhagiae (19.4%), Bratislava (8.5%), Grippotyphosa (7.2%), and Canicola (3.4%) have also been described. However, a significant bias of these studies is that the majority of dogs (*n =* 85) were vaccinated, and thus positive sera could also have been the result of a possible cross-reaction commonly linked to post-vaccination.

Eighty-seven sera of dogs (53%) that had been recently vaccinated (six months before the sample collection) showed low titers of antibodies against Icterohaemorrhagiae (*n =* 41), Copenhageni (*n =* 45), and Canicola (*n =* 16; 19% (≥1:100/1:200) serovars, which are included in the bivalent vaccine formulation. However, one vaccinated dog showed a seropositivity (MAT ≥ 1:3200) against Icterohaemorrhagiae serovar, and we hypothesized an absence of immune response after vaccine administration. Another important aspect linked to this result is that vaccination should be administered in non-infected dogs. When dogs are already infected, vaccination cannot prevent the disease. Although vaccination should act as available option to decrease the spread of the infection, standard hygiene measures before and after the contact with potentially infected animals or contaminated water should be taken to prevent dissemination of *Leptospira* in the environment and between animals.

Seropositive cases recorded against serovar that are not included in vaccine composition such as Bratislava (titers from 1:100 to 1:800) and Grippotyphosa (1:100 titer) could indicate a recent exposure to *Leptospira* infection. This also could be interpretated as cross-reactivity to nonvaccine serovars, demonstrating that MAT titers are not serovar specific. According to other studies [51], these results indicate that vaccination used until now is not always effective to dog immunization since the number of serovars involved in *Leptospira* infections and circulating among animals is very high, and the epidemiology of human infections always reflects the circulation of the bacterium in animal reservoirs. Dogs that can shed leptospires without being symptomatic represent a possible risk for humans and other animals [17]. The real-time PCR (qPCR) targeting the *lipL32* gene conducted on urine samples collected from 18 dogs showed low diagnostic sensitivity, and no positive results were obtained. PCR results are usually less sensitive when compared to the serological reactions because molecular results depend on the phase of the diseases, as well as potentially being related to the low concentration of leptospires in urine. Moreover, PCR-based diagnosis does not allow for identification of the circulating serovars in a particular geographical area. However, further studies are needed to better isolate *Leptospira* strains by collecting more dog urine samples and assessing whether dogs are shedding the pathogen in the environment. Wild rodents represent the main reservoir of leptospires, and the epidemiology of *Leptospira* infections reflects the circulation of the bacterium in animal reservoirs [52]. Out of 237 kidney samples from rodents, 7 were successfully cultured, illustrating the difficulty of the diagnosis of *Leptospira* by the standard routine laboratory culture techniques and the importance of development of new easy techniques that could allow better diagnosis of leptospirosis. Results obtained from the seven isolates from rat kidneys with MLST allowed for the characterization of the *Leptospira* strains by sequencing and analyzing specific fragments of some bacterial housekeeping genes, thus identifying specific sequence types (STs). These results were in agreement with the recent detection of pathogenic *Leptospira* from wild mammals [24] in the same study area and indicate that serovar diversity of *Leptospira* species has yet to be fully investigated in Sardinia Island.

## 5. Conclusions

Despite the role of dogs as possible source of human infection and their contribution to the circulation of *Leptospira* in the environment having been widely reported worldwide [34,35,46,53], cases of dog–human transmission have not yet been reported in the study area. However, detection of pathogenic *Leptospira* serovars in dogs from North Sardinia highlights that dogs represent a potential infection risk for people and contribute to bacteria spread in the environment. Public health strategies for controlling possible outbreak of *Leptospira* should be implemented in order to avoid the disease becoming a major medical and veterinary problem in this region.

## Figures and Tables

**Table 1:** Summary of dogs and rodents analyzed in this study, sites where kennels were located, and relative number of captured rodents.

Collection of Dogs	Kennel Location (Abbreviation Letter)	No. of Hosted Dogs	No. of Tested Dogs	No. of Captured Rodents
	Ossi (A)	130	63	30
	Alghero (B)	450	298	48
	Porto Torres (C)	80	41	12
	Codrongianos (D)	130	100	22
	Porto Torres (E)	100	50	27
Kennel	Sassari (F)	280	149	36
	Porto Torres (G)	100	49	11
	Ittiri (H)	320	194	15
	Sassari (I)	55	31	7
	Sassari (L)	280	160	15
	Sassari (M)	150	73	14
Private	Sassari	-	88	-
Total	11	2075	1296	237

**Table 2:** Genomospecies, serovar, strain, and serogroup of *Leptospira* used as antigens for the microscopic agglutination test.

Genomospecies	Serogroup	Serovar	Strain
*L. interrogans*	Sejroe	Hardjo	Hardjoprajitno n°224
	Australis	Bratislava	Hedgehog n°47
	Pomona	Pomona	Pomona n°222
	Icterohaem.	Icterohaem.	RGA 20
	Icterohaem.	Copenhageni	Wijnberg n°1
	Canicola	Canicola	Alarik n°2
*L. kirschneri*	Grippotyphosa	Grippotyphosa	Moska V n°54
*L. borgpetersenii*	Tarassovi	Tarassovi	Mitis Johnson n°6
	Ballum	Ballum	Mus 127 n°217

**Table 3:** Summary of MAT-positive dogs analyzed in this study.

Kennels	MAT Analysis	MAT Positives ($\overset{-}{x}$; 95% CI)
A	63	20 (32; 20–44)
B	298	4 (1; 0–2)
C	41	9 (22; 9–35)
D	100	8 (8; 3–13)
E	50	7 (14; 4–24)
F	149	34 (23; 16–30)
G	49	1 (2; 0–6)
H	194	32 (17; 12–22)
I	31	0
L	160	29 (18; 12–24)
M	73	10 (14; 6–22)
Owned	88	10 (11; 4–18)
Total	1296	164 (13%; 11–15)

**Table 4:** Prevalence of positive MAT results in the dogs’ population divided by kennel location sex, age, size, and vaccination status.

Dog Information	Kennel Dogs											Owned Dogs	Tot.
	A	B	C	D	E	F	G	H	I	L	M		
Sex														
Male	9	3	6	2	2	17	0	20	0	12	6	3	80
Female	11	1	3	6	5	17	1	12	0	17	4	7	84
Total	20	4	9	8	7	34	1	32	0	2	9	10	164
**Age**														
<2	0	0	0	1	0	0	0	1	0	9	0	1	12
2–8	8	3	5	6	0	22	1	24	0	12	6	9	96
>8	12	1	4	1	7	12	0	7	0	8	4		56
Total	20	4	9	8	7	34	1	32	0	29	10	10	164
**Size**														
Large	11	0	1	4	0	4	0	5	0	2	1	1	29
Medium	1	2	8	3	3	24	1	27	0	26	9	6	110
Small	8	2	0	1	4	6	0	0	0	1	0	3	25
Total	20	4	9	8	7	34	1	32	0	29	10	10	164
**Vaccination status**														
No	20	0	4	0	0	0	0	24	0	11	10	8	77
<6 months	0	4	5	8	7	34	1	8	0	18	0	2	87
Total	20	4	9	8	7	34	1	32	0	29	10	10	164

**Table 5:** Number of positive microscopic agglutination test (MAT) results among dogs never vaccinated and vaccinated from less than six months.

Genospecie	Serogroup	Serovar	Number of Dogs with Respective MAT Titers													
			1:100		1:200		1:400		1:800		1:1600		1:3200			
			Vaccination in Months												Total (%)	
			No	<6	No	<6	No	<6	No	<6	No	<6	No	<6	No	<6
*L. interrogans*	Australis	Bratislava	5	13	5	-	2	4	-	2	1	-	-	-	13 (17)	19 (22)
*L. interrogans*	Canicola	Canicola	3	16	3	-	-	1	-	-	-	-	-	-	6(8)	17 (20)
*L. interrogans*	Icterohaem.	Copenhageni	30	45	14	-	2	2	-	-	1	-	-	-	47 (61)	47 (54)
*L. interrogans*	Icterohaem.	Icterohaem.	20	41	16	-	8	6	2	-	-	-	1	1	47 (61)	48 (55)
*L. kirschneri*	Grippotyphosa	Grippotyphosa	5	6	1	-	-	-	-	-	-	-	-	-	6 (8)	6 (7)

**Table 6:** Results of serology by MAT, qPCR, culture assay, and MLST performed on samples collected from rodents.

Rodent Group	Scientific Name (*n*.)	Source	MAT		qPCR		Cultural Isolation		MLST (*n*.)
			Tested	Positives (%; 95%CI)	Tested	Positives (%; 95%CI)	Tested	Positives (%;95%CI)	
Rat (160)	*Rattus rattus* (158)	Kidney	-	-	158	13 (8; 4–12)	158	3 (2; 0–4)	ST17 (1); ST 149 (2)
		Liver	-	-	3	1 (33; 0–83)	3	0	
		Urine	-	-	2	0	2	0	
		Serum	15	0	-	-	-	-	
	*Rattus norvegicus* (2)	Kidney	-	-	2	1 (50; 0–120)	2	1 (50;	ST 198 (1)
		Liver	-	-	1	0	1	0	
		Urine	-	-	1	0	1	0	
		Serum	2	0	-	-	-	-	
Mouse (77)	*Apodemus sylvaticus* (70)	Kidney	-	-	70	9 (13; 5–21)	70	3 (4; 0–9)	ST 149 (3)
		Liver	-	-	2	1 (50; 0–120)	2	0	
		Urine	-	-	1	0	1	0	
	*Mus musculus* (7)	Kidney	-	-	7	2 (29; 18–40)	7	0	
		Liver	-	-	1	1 (100)	1	0	
Total	237		17	0	237	28 (12; 8–16)	237	7 (3; 1–5)	

## Data Availability

The data presented in this study are available in the article.
